# Swim training affects Akt signaling and ameliorates loss of skeletal muscle mass in a mouse model of amyotrophic lateral sclerosis

**DOI:** 10.1038/s41598-021-00319-1

**Published:** 2021-10-22

**Authors:** Karol Cieminski, Damian Jozef Flis, Katarzyna Dzik, Jan Jacek Kaczor, Emilia Czyrko, Malgorzata Halon-Golabek, Mariusz Roman Wieckowski, Jedrzej Antosiewicz, Wieslaw Ziolkowski

**Affiliations:** 1 Poznan University of Physical Education, Poznan, Poland; 2 Department of Physiology and Biochemistry, Gdansk University of Physical Education and Sport, 80-336 Gdansk, Poland; 3 Department of Pharmaceutical Pathophysiology, Faculty of Pharmacy, Medical University of Gdansk, Gdansk, Poland; 4 Department of Animal and Human Physiology, Faculty of Biology, University of Gdansk, Gdansk, Poland; 5 Department of Physiotherapy, Faculty of Health Sciences Medical, University of Gdansk, Gdansk, Poland; 6 Laboratory of Mitochondrial Biology and Metabolism, Nencki Institute of Experimental Biology, Warsaw, Poland; 7 Department of Bioenergetics and Physiology of Exercise, Faculty of Health Sciences, Medical University of Gdansk, Gdansk, Poland; 8 Department of Rehabilitation Medicine, Faculty of Health Sciences Medical, University of Gdansk, 80-219 Gdansk, Poland

**Keywords:** Molecular biology, Neurodegeneration

## Abstract

We tested the hypothesis that swim training reverses the impairment of Akt/FOXO3a signaling, ameliorating muscle atrophy in ALS mice. Transgenic male mice B6SJL-Tg (SOD1^G93A^) 1Gur/J were used as the ALS model (*n* = 35), with wild-type B6SJL (WT) mice as controls (*n* = 7). ALS mice were analyzed before ALS onset, at ALS onset, and at terminal ALS. Levels of insulin/Akt signaling pathway proteins were determined, and the body and tibialis anterior muscle mass and plasma creatine kinase. Significantly increased levels of FOXO3a in ALS groups (from about 13 to 21-fold) compared to WT mice were observed. MuRF1 levels in the ONSET untrained group (12.0 ± 1.7 AU) were significantly higher than in WT mice (1.12 ± 0.2 AU) and in the BEFORE ALS group (3.7 ± 0.9 AU). This was associated with body mass and skeletal muscle mass reduction. Swim training significantly ameliorated the reduction of skeletal muscle mass in both TERMINAL groups (p < 0.001) and partially reversed changes in the levels of Akt signaling pathway proteins. These findings shed light on the swimming-induced attenuation of skeletal muscle atrophy in ALS with possible practical implications for anti-cachexia approaches.

## Introduction

Amyotrophic lateral sclerosis (ALS) is an incurable, chronic neurodegenerative disease characterized by selective death of motoneurons, which control any muscle activity in the motor cortex, brainstem, and spinal cord^1^. ALS is phenotypically characterized by the loss of muscle tone, paresis, muscle atrophy, and spasticity^2^. Approximately 90% of ALS cases are sporadic (sALS), with unknown etiology, and the rest are genetically determined (fALS). In clinical terms, both forms are identical.

The role of muscle in ALS has been extensively studied and gave rise to highly inconsistent results. On the one hand, the data from the last decade shows that the causes of neurodegeneration in ALS should be sought outside the nervous system. For example, overexpression of SOD1^G93^^A^ in skeletal muscle initiates motoneurons death^2^ and causes profound muscle atrophy^2,3^. Additional support for this view is provided by the observations that the destruction of neuromuscular junctions has been linked to oxidative stress induced by a tissue-specific breakdown of muscle mitochondria^4^. Nevertheless, on the other hand, no evidence targeting muscle to boost its volume/function provides compelling, lasting, or meaningful protective effects against motoneurons degeneration and clinical motor deficits in rodent models or even patients.

The imbalance between muscle protein synthesis and proteolysis in ALS results in muscle atrophy and is regulated by the insulin/Akt signaling pathway^5,6^. The insulin/Akt/Forkhead box O3 (FOXO3a) signaling pathway is essential for protein breakdown and autophagy, while the insulin/Akt/mammalian target of rapamycin (mTOR) signaling pathway plays an essential role in muscle protein synthesis and regeneration^7^. Activation or deactivation of the Akt serine/threonine kinase determines the course of these pathways. Active FOXO3a protein is responsible for stimulating muscle protein breakdown and increasing antioxidant defense enzyme activity^5,7^. We have recently shown that Akt is inactivated, while FOXO3a is activated, with ALS development in rat skeletal muscle, accompanied by an increased expression of Atrogin-1, a protein responsible for muscle cell atrophy^5^. Of note, despite the accompanying increased activity of FOXO3a-stimulated antioxidant enzymes, oxidative stress in ALS is higher than that in wild-type (WT) skeletal muscles^5,8–10^. We have also demonstrated that changes in the active forms of Akt and FOXO3a are responsible for ferritin up-regulation and iron accumulation in skeletal muscle of ALS rat and are accompanied by an increase in amyloid precursor protein and iron chaperone PCBP1 levels^5^.

An increasing number of studies suggest that swim training prolongs the lifespan of ALS mice, with an accompanying improvement in muscle bioenergetics and glucose metabolism and a reduction of muscle fiber loss and weight^11–13^. In addition, recently, we have shown that swim training attenuates the reduction of muscle strength in ALS mice^14^ and positively influences skeletal muscle mitochondria's function by modifying the cellular structure components created by the mitochondria and endoplasmic reticulum membranes^13^.

Swim training in WT animals leads to the activation of Akt and mTOR proteins^15^. To the best of our knowledge, the impact of swim training on the Akt signaling pathway and the accompanying process of muscle atrophy has not been evaluated to date. Therefore, we hypothesized that ALS modulates the insulin/Akt/FOXO3a pathway, resulting in muscle atrophy, and that swim training at least partially reduces aberrant activation of this pathway in ALS, with a concomitant amelioration of skeletal muscle mass atrophy. We analysed representative proteins of the insulin/Akt/mTOR and insulin/Akt/FOXO3a pathways in the ALS mouse model, with and without training, and at different ALS stages to test this hypothesis.

## Results

### Clinical score of the ALS mice at sacrifice time

On the day of euthanasia, mice in the ALS BEFORE, ONSET, and TERMINAL untrained groups had clinical scores of 0, 1, and 4.5 ± 0.2 points, respectively. On the same day, mice from trained groups had a clinical score of 0.4 ± 0.2 and 2.8 ± 0.7 points in the ONSET and TERMINAL groups, respectively.

### The effect of swim training on the Akt signaling pathway in ALS skeletal muscle

#### IGF-1/Akt signaling pathway proteins

**Figure 1:**
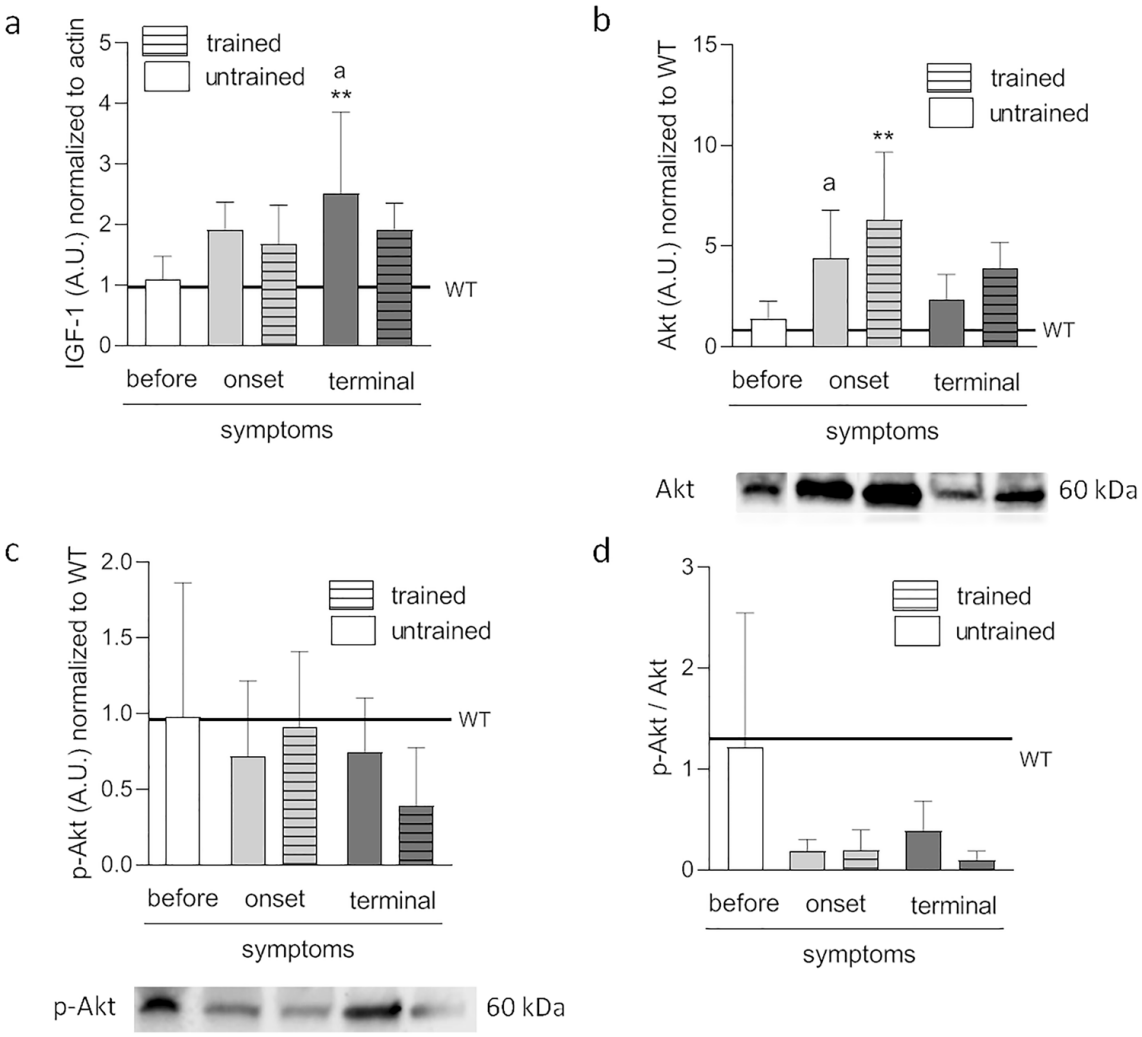
Effects of ALS disease progression and swim training on IGF-1 and Akt signaling pathway. IGF-1 **(a)**, Akt **(b)**, p-Akt **(c)**, and p-Akt to Akt ratio **(d)** were measured in tibialis anterior muscle. There were significant differences between the groups: **p < 0.01 vs. WT group of mice, ^a^p < 0.05 vs. BEFORE group, The data are presented as the means ± SEM (n = 5 in each group). Full-length blots are displayed in Supplementary Fig. S6 and Fig. S7.

IGF-1 levels were significantly higher in untrained TERMINAL mice than in WT mice (2.52 ± 0.5 AU). Further, IGF-1 levels were significantly higher in the TERMINAL group than in the BEFORE group (1.1 ± 0.2 AU) (Fig. 1a).

Akt levels were significantly higher in trained ALS mice at the ONSET stage of the disease than in WT mice (6.3 ± 1.5 AU). In the ONSET untrained group, Akt levels were significantly higher than those in the BEFORE group (4.4 ± 1.1 and 1.4 ± 0.4 AU, respectively). At terminal-stage disease, swim training increased Akt level (2.4 ± 0.5 and 3.9 ± 0.6 AU in TERMINAL untrained and trained, respectively; Fig. 1b).

No significant changes in the active form of Akt (p-Akt) and p-Akt/Akt ratio were apparent between the groups (Fig. 1c,d). The p-Akt/Akt ratios in the ONSET and TERMINAL groups were greatly reduced compared with BEFORE and WT groups, but the change was not statistically significant.

Changes in the IGF-1 and Akt protein levels described above were confirmed by the analysis of tissue proteomes (Fig. S2).

#### Akt-dependent signaling pathway proteins responsible for the proteolysis of muscle proteins

**Figure 2:**
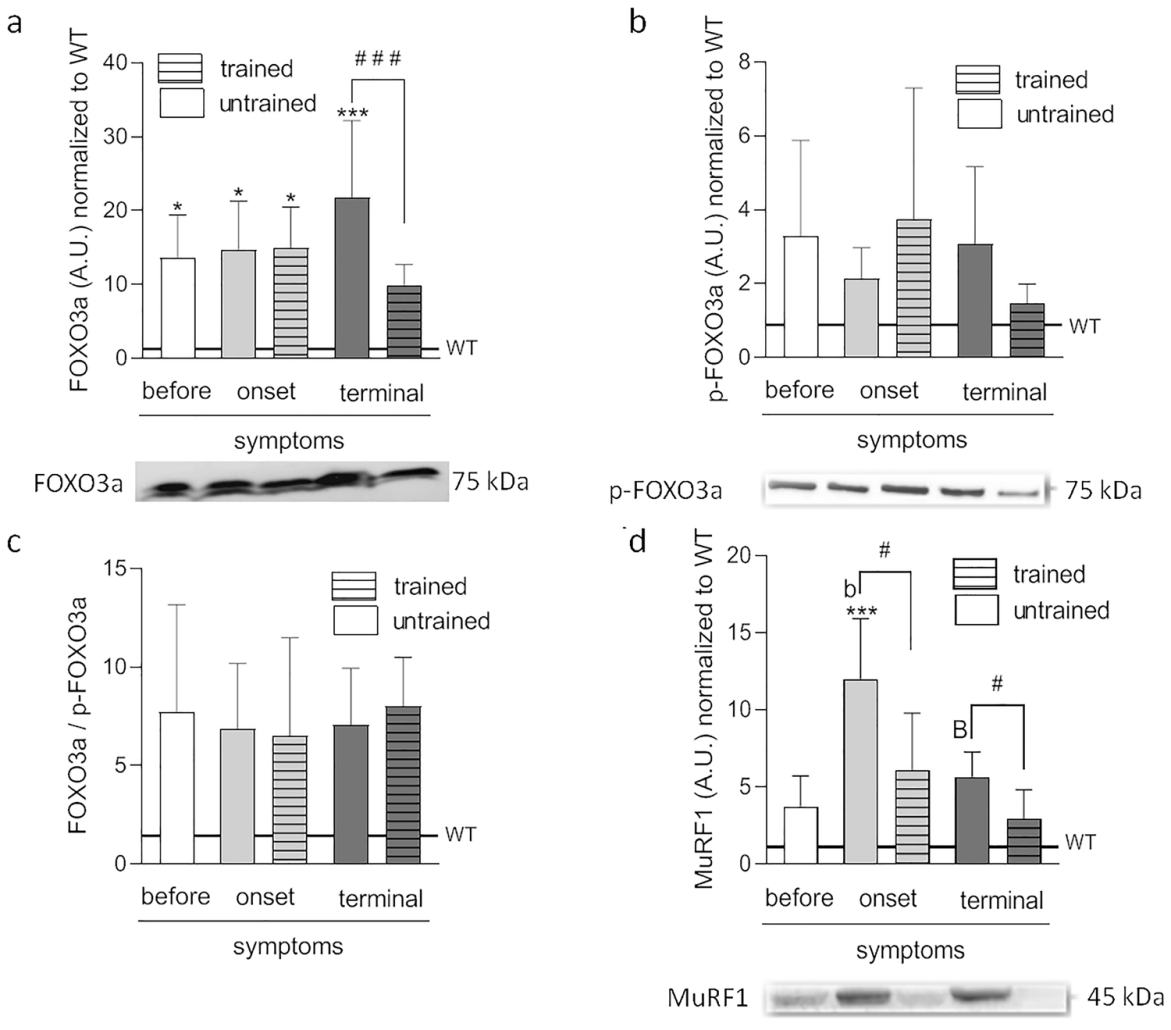
Effects of ALS disease progression and swim training on the proteins responsible for the proteolysis of muscle proteins. FOXO3a **(a)**, p-FOXO3a **(b)**, FOXO3a to p-FOXO3a ratio **(c)**, and MuRF1 **(d)** were measured in tibialis anterior muscle. There were significant differences between the groups: *p < 0.05, ***p < 0.001 vs. WT group of mice, ^#^p < 0.05, ^###^p < 0.001 between indicated group, ^b^p < 0.01 vs. BEFORE group, ^B^p < 0.01 vs. ONSET group, The data are presented as the means ± SEM (n = 5 in each group). Full-length blots are displayed in Supplementary Fig. S8, Fig S9 and Fig. S10.

FOXO3a and p-FOXO3a levels, and FOXO3a/p-FOXO3a ratio were next analyzed. A significant increase in the active form of FOXO3a in the ALS BEFORE, and the ONSET untrained and the ONSET trained, and TERMINAL untrained groups (13.6 ± 2.6, 14.7 ± 2.9, 14.9 ± 2.5, and 21.8 ± 4.6 AU, respectively) in comparison with WT mice (1.29 ± 0.22 AU) was observed. At terminal-stage disease, swim training induced FOXO3a level reduction (9.9 ± 1.2 AU) (Fig. 2a). However, no significant differences between the groups were apparent concerning the inactive FOXO3a (p-FOXO3a) levels and the FOXO3a/p-FOXO3a ratio (Fig. 2b,c).

MuRF1 and Atrogin-1 induce proteolysis in skeletal muscle cells. However, the observed changes in the levels of these two proteins were not the same. Specifically, MuRF1 levels in the ONSET untrained group were significantly higher than in WT mice (12.0 ± 1.7 AU). In the untrained ONSET group, MuRF1 levels were higher than those in the BEFORE ALS group (3.7 ± 0.9 AU). An increase in MuRF1 levels accompanied ALS progression. At terminal-stage disease in the untrained group, MuRF1 levels were reduced (5.6 ± 0.7 AU) compared with those in the untrained ONSET group. Further, swim training induced MuRF1 level reduction in the ONSET and TERMINAL groups (6.1 ± 1.7 and 1.9 ± 0.8 AU, respectively) (Fig. 2d). By contrast, no significant changes in Atrogin-1 levels were apparent in any analyzed groups (Fig. S3).

Proteome analysis of FOXO3a, Atrogin-1, and MuRF1 levels in ALS BEFORE, and TERMINAL trained and untrained groups confirmed the above changes (Fig. S4).

#### Proteins involved in the synthesis of muscle proteins

**Figure 3:**
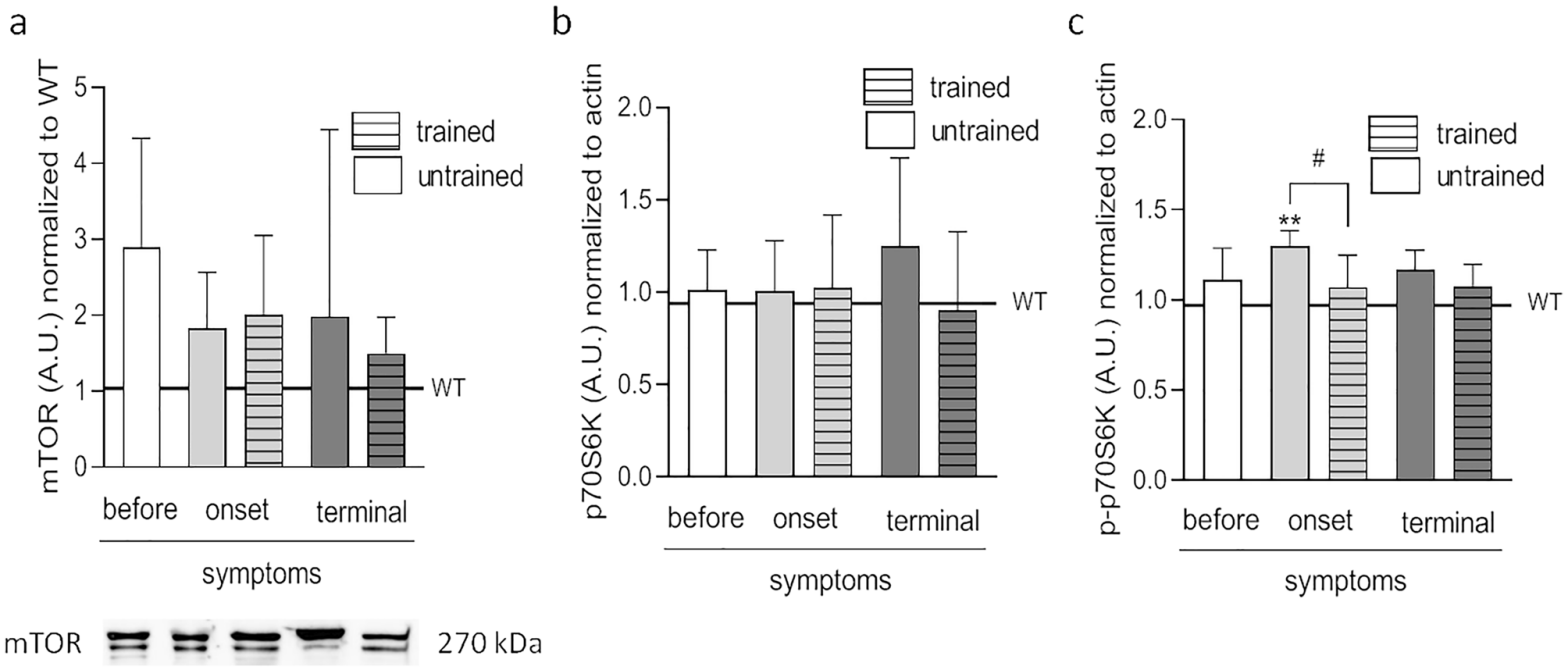
Effects of ALS disease progression and swim training on the proteins involved in the process of muscle protein synthesis. mTOR **(a)**, p70S6K **(b)**, p-p70S6K **(c)** were measured in the tibialis anterior muscle. There were significant differences between the groups: **p < 0.01 vs. WT group of mice, ^#^p < 0.05 between indicated group, The data are presented as the means ± SEM (n = 5 in each group). Full-length blot is displayed in Supplementary Fig. S11.

No changes in the mTOR levels were noted in any of the analyzed groups (Fig. 3a). Further, p70S6K levels were unaffected by ALS or swim training (Fig. 3b). Interestingly, in the untrained ONSET group, levels of the active form of p70S6K (p-p70S6K) (1.3 ± 0.03 AU) were higher than those in WT mice. Further, at the onset of disease symptoms, swim training significantly reduced p-p70S6K levels (1.1 ± 0.1 AU vs. untrained animals) (Fig. 3c).

The proteomic analysis confirmed the lack of a clear directionality of changes in the levels of anabolic proteins in trained TERMINAL vs. untrained TERMINAL and BEFORE ALS mice (Fig. S5).

### The effect of swim training on skeletal muscle mass in ALS mice

**Figure 4:**
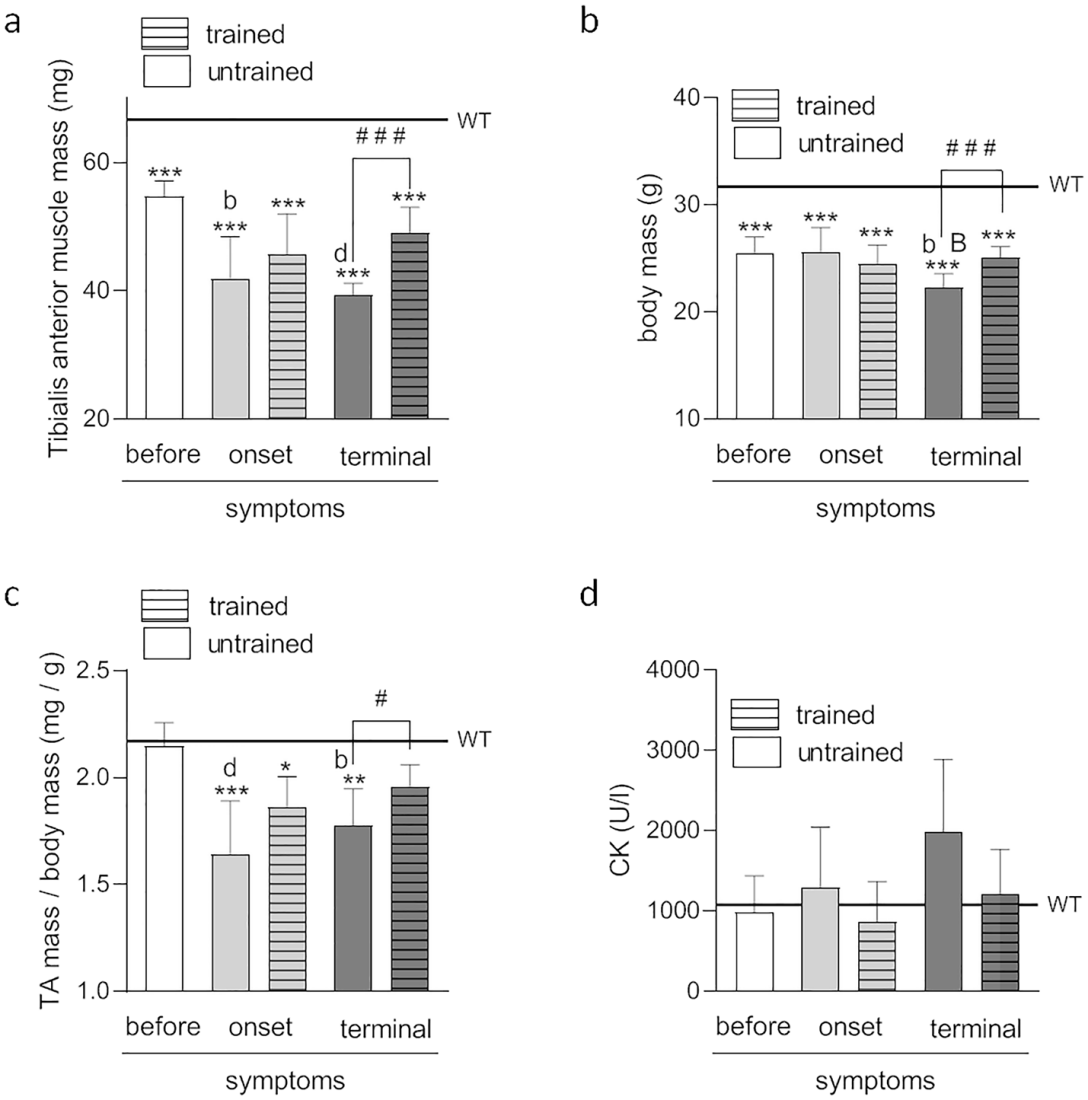
Effects of ALS disease progression and swim training on body mass, tibialis anterior muscle mass, and blood creatine kinase activity. Tibialis anterior muscle mass **(a)** body mass **(b)**, the ratio of tibialis anterior to body mass **(c)**, and activity of blood creatine kinase **(d)** in mice. There were significant differences between the groups: *p < 0.05, **p < 0.01, ***p < 0.001 vs. WT group of mice, ^#^p < 0.05, ^###^p < 0.001 between indicated group, ^b^p < 0.01, ^d^p < 0.001 vs. BEFORE group, ^B^p < 0.01 vs. ONSET group, The data are presented as the means ± SEM [n = 7 in each group (a,b,c) and n = 6 in each group (d)].

ALS reduced TA muscle mass in all ALS mice compared to WT mice (68.7 ± 1.4 mg), with a reduction in skeletal muscle mass related to disease progression (54.8 ± 0.9, 41.9 ± 2.4, and 39.3 ± 0.7 mg in ALS BEFORE, ONSET, and TERMINAL groups, respectively). Swim training ameliorated the reduction in skeletal muscle mass (45.7 ± 2.4 and 49.1 ± 1.5 mg in the trained ONSET and TERMINAL groups, respectively) (Fig. 4a). Besides skeletal muscle mass reduction, ALS also affected mouse body mass so that the body mass of ALS mice was lower than that of WT mice (31.7 ± 0.7 g). The body mass of animals in the TERMINAL groups (22.2 ± 0.5 g) was significantly lower than in the BEFORE and ONSET groups (25.5 ± 0.5 and 25.6 ± 0.8 g, respectively). In addition, swim training attenuated body mass reduction at terminal-stage disease (25.0 ± 0.4 g) (Fig. 4b).

To evaluate skeletal muscle atrophy, the ratio of TA to animal body mass was calculated. In comparison to WT mice (2.2 ± 0.1 mg/g), the ratio was significantly reduced in the untrained ONSET, trained ONSET, and untrained TERMINAL groups (1.6 ± 0.04, 1.9 ± 0.05 and 1.8 ± 0.04 mg/g, respectively). The ratio was significantly higher in the TERMINAL trained group (2.0 ± 0.04 mg/g) than in the TERMINAL untrained group (Fig. 4c).

### The effect of swim training on skeletal muscle damage in ALS mice

No significant differences in plasma CK activity were observed between the groups (Fig. 4d). However, a tendency (p = 0.078 BEFORE vs. untrained TERMINAL) of increasing plasma CK activity during disease progression was apparent (982.2 ± 186.3, 1295.0 ± 306.4, and 1983.0 ± 369.9 U/L in the untrained ALS BEFORE, ONSET, and TERMINAL groups, respectively). Conversely, swimming reduced the CK activity in the trained ONSET and TERMINAL groups (860.0 ± 207.1 and 1206.0 ± 229.5 U/L, respectively), but the effect was not significant (Fig. 4d).

## Discussion

We showed that swim training exerts an anti-atrophic effect by influencing the levels of Akt signaling pathway proteins, particularly the Foxo-3/MuRF1 proteins. Muscle atrophy is an active process controlled by specific signaling pathways and transcriptional programs. Indeed, the genes induced most strongly in muscle atrophy encode two muscle-specific ubiquitin ligases, Atrogin-1 (also known as MAFbx) and MuRF1^16,17^. The obtained results confirmed our hypothesis that ALS progression modulates the insulin/Akt/FOXO3a pathway, resulting in muscle atrophy, and that swim training partially reduces the aberrant activation of this pathway, with a concomitant amelioration of skeletal muscle mass atrophy.

The insulin/Akt signaling pathway begins with the common IGF-1–Akt axis. IGF-1, a circulating growth factor, is produced locally by many tissues, including skeletal muscle (reviewed in^18^). IGF-1 sustains muscle growth and regeneration^19^, and IGF-1 and/or insulin signaling suppresses protein breakdown while promoting muscle growth. Furthermore, IGF-1 plays a crucial role in the inhibition of muscle atrophy and cardiac cachexia. We observed a progressive increase in muscle IGF-1 levels accompanying ALS development, which could be explained as a 'muscle's defense mechanism against atrophy. Swim training did not affect IGF-1 levels.

The binding of IGF-1 to its receptor triggers the activation of several intracellular kinases, including phosphatidylinositol-3-kinase (PI3K). PI3K phosphorylates the membrane phospholipid phosphatidylinositol-4,5-bisphosphate to phosphatidylinositol-3,4,5-trisphosphate (PIP3), creating a lipid-binding site on the cell membrane for the serine/threonine kinase Akt (also PKB, protein kinase B). The subsequent translocation of Akt to the membrane facilitates its phosphorylation and activation by the PDK-1 kinase. Therefore, we evaluated the total and active forms of Akt in skeletal muscle of ALS mice and the effect of swim training on Akt activation. Unlike the rising IGF-1 levels, the p-Akt/Akt ratio decreased during disease. Considering our previous studies in a rat ALS model^5^ and other reports^20^, these observations confirm Akt inactivation during ALS progression.

A possible explanation of the above phenomenon is inhibiting IGF-dependent signaling by phosphatase and tensin homolog (PTEN). PTEN dephosphorylates PIP3 and thus inhibits its activity, resulting in the inhibition of Akt. Further, oxidative stress leads to the activation of PTEN phosphatase, which dephosphorylates the IGF-1 receptor and PI3K, dampening IGF-1 signaling^21^. Therefore, PTEN involvement in ALS is plausible, as we have previously reported on the occurrence of oxidative stress in the muscle of ALS mouse^13,14^. Nonetheless, this possibility requires further confirmation.

Although no statistically significant differences were noted in the current study, the p-Akt/total Akt ratio in the ONSET and TERMINAL groups was lower than that in the BEFORE and WT groups, indicating inactivation of this protein in the former two groups. Transgenic mice overexpressing Akt in skeletal muscle display muscle hypertrophy and protection from denervation-induced atrophy^22,23^, confirming that the Akt pathway promotes muscle growth and simultaneously blocks protein degradation^22,24^. We showed that swim training increases the total Akt protein in the TA muscle of ALS mice in the ONSET and TERMINAL groups, which was additionally illustrated by proteome analysis of the IGF-1 and Akt protein levels (Fig. S2), and could be considered as a protective phenomenon. However, this did not translate into an increased fraction of the active form of Akt in animals suffering from ALS, which further should affect the activity of the FOXO3a protein. Consequently, we measured the expression of FOXO3a and its downstream targets, i.e., Atrogin-1 and MuRF1, in skeletal muscle. The FOXO3a/Atrogin-1/MuRF1 signaling pathway is involved in the induction of muscle protein proteolysis and muscle atrophy^7^. As anticipated, levels of FOXO3a and p-FOXO3a, and the FOXO3a/p-FOXO3a ratio, indicative of FOXO3a activation, were increased in the TA muscle in ALS mice compared with the controls. At the same time, swim training decreased the FOXO3a and p-FOXO3a levels (the latter, without statistical significance) in the TERMINAL group. This was accompanied by changes in MuRF1 levels in the ONSET and TERMINAL groups. Interestingly, in contrast to the results of our studies in the rat ALS model and other reports^5,20^, we did not observe changes in the Atrogin-1 levels in ALS animals, despite the increase in the FOXO3a levels (Fig. S3). The same was previously reported by Dobrovolny^3^. Atrogin-1 can control both the processes associated with the breakdown of cytoskeletal proteins and processes associated with protein synthesis, while MuRF1 is involved only in the breakdown of myofibrillar proteins. Due to these facts, there may be another mechanism in skeletal muscle that regulates the expression of atrogin-1 independent of phosphorylation/dephosphorylation of FOXO3a, like histone deacetylase-1^25^. Changes in the FOXO3a and MuRF1 expression can be explained by the notion that swim training reduces oxidative stress in the muscles of animals with ALS^13^. Growing evidence suggests that increased reactive oxygen species production in skeletal muscle significantly induces mitochondrial dysfunction, activates forkhead box class O (FoxO) transcription factors, and contributes to muscle atrophy^26,27^. In the current study, the proteome analysis of the FOXO3a, Atrogin-1, and MuRF1 levels in ALS mice revealed a significant shift in the expression of these proteins in the TERMINAL trained group towards the BEFORE group levels, which further confirms the effectiveness of swim training as an anti-atrophic agent.

Apart from FOXO3a, the second target of Akt is mTOR, which, together with p70S6K, is responsible for the synthesis of muscle proteins^28^. We did not observe any significant differences in these protein levels in the studied groups of animals in the current study. Further, swim training resulted in a decrease of p-p70S6K levels only in the ONSET group. Thus, the effect of swim training in the ONSET group is difficult to explain. However, in one 2018 study, inhibition of p70S6K by A77 1726, the active metabolite of the anti-inflammatory drug leflunomide, induced mTOR feedback activation and UNC-51-like kinase 1 phosphorylation in NSC34 cells, a hybrid mouse motoneuron cell line^29^. This was accompanied by an induction of co-localization of SOD1 G93A variant aggregates with autophagosomes and accelerated SOD1 G93A degradation. This suggests that p70S6K inhibition induces autophagy in NSC34 cells and that blocking S6K1 activity by a small molecule inhibitor, such as leflunomide, may offer a new strategy for ALS treatment^29^. However, whether the effect of swim training is based on the same mechanism remains to be clarified.

In another study^30^ no difference in p70 ribosomal S6 kinase (S6K) activity in ALS patients and controls was reported in the spinal cord tissue. However, the amount of S6K protein in ALS patients was significantly higher than in the controls^30^. In yet another study^20^, analysis of skeletal muscle biopsies from individuals with ALS and controls revealed no differences in p70S6K expression on the mRNA and protein levels, although the Akt activity was relatively lower in the ALS group. Thus, no changes in the expression of p70S6K may result from a decreased activation of the Akt protein in the muscle of ALS animals.

Further, mTOR kinase 'levels' did not significantly change in the ALS skeletal muscle. To the best of our knowledge, this is the first report on mTOR protein levels in skeletal muscle in ALS mice. Similar trends in mTOR expression were reported earlier in ALS cortical neuron cells ^31^. Proteome analysis confirmed the lack of changes in these protein levels (Fig. S5). The above observations indicate that the anti-atrophic effect of swim training is based on the inhibition of the FOXO3a/MuRF1 pathway and not on protein induction of the mTOR/p70S6K pathway. This agrees with the notion that disruption of the balance between protein synthesis and catabolism leads to muscle atrophy^5,6^.

ALS causes progressive muscle atrophy, ultimately leading to death^32^. Accordingly, in the current study, ALS (SOD1 G93A) mice showed progressive muscle loss accompanied by weight loss. This confirmed earlier observations^9,33^. In addition, to determine whether the muscle and body mass loss is accompanied by sarcolemma breakdown and muscle cell damage, we determined CK activity in the plasma of ALS mice. Changes in blood CK levels are related to general skeletal muscle cells damage associated with hypertrophy of remaining fibers as a result of the progressive secondary myopathy^34^. The CK activity was elevated in the ALS TERMINAL untrained group as compared to other groups. However, the differences were not statistically significant.

Interestingly, plasma CK values are significantly elevated in most, but not all, ALS patients. For example, in one study, CK activities were elevated in 43% of patients (mean, 240 U/L; range, 59–1327 U/L). However, elevated CK levels did correlate with spinal somatic muscle weakness in that study. Ninety-three percent of patients with elevated CK levels observed spinal somatic muscle weakness as their major presenting symptom^35^. There is also evidence of elevated serum CK levels in the early stage of sALS^36^. Changes in the blood CK activities in patients with ALS were recently presented in a new perspective, with higher CK levels reported for an ALS model with a slowly progressing disease than in an ALS model with a fast-progressing disease^37^. The study suggested that the slow-progressing mice progress slower because they have greater muscle mass and may counter disease mechanisms for more extended periods than the fast-progressing mice^37^.

In the current study, we demonstrated that swim training reduces weight and muscle mass loss and is accompanied by insignificant drops in plasma CK activity. These changes were accompanied by an extended lifespan, with swim training reducing oxidative stress and improving the bioenergetics and muscle strength^13,14^.

The findings of our study have to be considered in light of some limitations. Although SOD1 G93A mice are still a gold standard in preclinical studies of ALS disease, additional studies are needed to confirm the obtained results on other ALS models. Moreover, the effects of swim training on the organism are multidimensional. Thus, the influence of swimming might occur through psychological, physiological, and molecular mechanisms that should be taken into account in future studies.

In conclusion, the presented data suggest that insulin signaling is impaired in the atrophied skeletal muscle in ALS mice, with concomitant induction of proteolytic protein levels. Swim training partially reversed these changes. These findings shed light on the swim-induced retardation of the development of skeletal muscle atrophy in ALS, with possible practical implications for anti-cachexia approaches.

## Methods

### Animals

All experimental procedures which included minimizing the number of animals and their suffering were reviewed and approved by the 3rd Local Ethical Committee for Experiments on Animals in Gdansk (decision number 11/2013, 22 April 2013) and the Polish Ministry of the Environment (decision number 155/2012, 05 December 2012). Guidelines for the handling, use, and ethical treatment of laboratory animals based on European Union Directive 2010/63/E.U. were followed in all experiments. The study was carried out in compliance with the ARRIVE guidelines.

Transgenic male mice expressing human SOD1 with the G93A substitution, B6SJL-Tg (SOD1G93A) 1Gur/J (ALS mice) (five groups, *n* = 7 per group) and WT male mice B6SJL (*n* = 7) were purchased from the Jackson Laboratory (Bar Harbor, ME, USA). The mice were housed in an environmentally controlled room (23 ± 1 °C, with a 12 h light/dark cycle) and received standard mice chow and water ad libitum. After acclimatization, the mice were randomly divided into the following groups, as previously described in^13,14^: ALS BEFORE, untrained ALS mice with no visible signs of the disease; ALS ONSET, mice with first symptoms of the disease, untrained and trained (see below); and ALS TERMINAL, animals at the last stage of the disease, untrained and trained. Clinical score assessment^38^ in the ALS mice was performed to establish the time of animals' scarification. Clinical score was assessed by an 8-point scale dependent on signs exhibited to identify the severity of the disease: 0 no evidence of disease; 1 shaking of the hindlimbs or splaying of the hindlimbs when suspended by the tail (an indication of weakness in the hindlimbs); 1.5 weakness in one hindlimb (compensation for footdrop); 2 change in gait; 2.5 extreme weakness in one hindlimb (inability to dorsiflex); 3 extreme weakness in both hindlimbs; 3.5 functional paralyzes in one hindlimb; 4 functional paralyzes in both hindlimbs but the animal can right itself in less than 20 s after being placed on its side; and 5 animal cannot right itself within 20 s after being placed on its side (endpoint, followed by euthanasia).

The mice were euthanized by cervical dislocation at the following time points: ALS BEFORE group at 10 weeks of age; ALS ONSET groups, at 16 weeks, when the first symptoms of the diseasewere apparent in untrained animals (clinical score—1); ALS TERMINAL groups, when the last stage of the diseasewas apparent in untrained ALS animals (clinical score—5) according to^13,38^.

### Swim training protocol

Starting at 70-day-old, animals in the ALS ONSET trained and ALS TERMINAL trained mouse groups underwent swim training, as described by Deforges et al.^11^, with slight modifications^13^. Briefly, the swim training was conducted in a special pool with regulated water flow five times a week for 30 min. The water temperature was 30 °C, and the maximum flow speed was 5 L/min. At 105-day-old, the training frequency was reduced to three times a week. The exercise time and water flow were adapted to the individual aptitude of the ALS groups. The training was terminated at 115-day-old.

### Body and tibialis anterior (TA) mass assessment

Body mass was assessed once a week in the morning. TA muscle samples were dissected at 4 °C at the end of the study and weighed to determine muscle atrophy. The data are presented as mg muscle per g of animal weight (mg/g)^9,33^.

### Tissue homogenization and lysate preparation

Following dissection, the TA muscle samples were frozen in liquid nitrogen and kept at –80 °C until analysis. The samples were homogenized using the Bio-Plex Pro ™ Cell Signaling Reagent kit (cat. no. 171-304006 M, Bio-Rad, Hercules, CA, USA), with minor modifications. Briefly, tissue (approximately 20 mg) was placed in a 1.5-mL Eppendorf tube and washed with 100 µL of Cell Wash Buffer. After rinsing and draining, the material was cut and transferred to a 2-mL Eppendorf tube. Then, 200 µL of Cell Lysis Buffer was added, and the sample was homogenized manually 20 times. The lysis buffer was prepared according to the 'manufacturer's instructions, but additionally contained a cocktail of protease (cat. no. P8340, Sigma Aldrich, St. Louis, MO, USA) and phosphatase inhibitors (PhosSTOP™, PHOSS-RO Sigma Aldrich,), 10 µL each per 1.02 mL of the final buffer volume. The lysate was then frozen at –70 °C and thawed at 30 °C, three times and re-homogenized 10 times. Finally, the material was centrifuged at 15,000×*g* for 10 min at 4 °C. The resulting supernatant was decanted and frozen at –70 °C for further analysis. Protein concentration in the lysate was determined using the Bradford method.

### Immunoblotting

Equal amounts of muscle lysates (50 µg of protein per sample) were separated on 4–20% SDS–polyacrylamide gradient gels and transferred onto a polyvinylidene difluoride membrane. The analysis procedure was performed according to^5^. The following antibodies were used: rabbit monoclonal IgG anti-p-FOXO3a (Ser253) (cat. no. ab154786, 1:1000; Abcam, Cambridge, Great Britain); rabbit polyclonal anti-p-Akt 1/2/3 (Ser473) (cat. no. sc-7985R, 1:1000; Santa Cruz Biotechnology, Dallas, TX, USA), anti-Akt 1/2/3 (cat. no. sc-8312, 1:600; Santa Cruz Biotechnology), anti-FOXO3a (cat. no. ab23683, 1:1000; Abcam), and anti-mTOR (cat. no. 2972, 1:1000; Cell Signaling, Beverly, MA, USA); mouse monoclonal IgG anti-MAFbx/Atrogin-1 (cat. no. sc166806, 1:1000; Santa Cruz Biotechnology) and anti-MuRF1 (cat. no. sc398608, 1:500; Santa Cruz Biotechnology). After washing (3 × 10 min) in 1 × TBST, the membranes were incubated for 1 h at room temperature with gentle shaking with anti**‐**rabbit IgG–peroxidase conjugate (cat. no. A9169, 1:25,000; Sigma Aldrich) and anti**‐**mouse IgG–peroxidase conjugate (cat. no. A9044, 1:25,000; Sigma Aldrich). After blocking in a blocking buffer as mentioned above, immunoblots were detected and visualized using enhanced chemiluminescence reagents (Pierce; Thermo Fisher Scientific, Inc., Waltham, MA, USA). Changes in protein levels were assessed by densitometry of the immunoreactive bands and normalized to the total amount of protein in the samples transferred onto the membrane. Relative protein levels were analyzed and quantified using ChemiDoc image analysis system (Bio**‐**Rad Laboratories, Inc.), as shown in Fig. S1 ALS group data were then normalized to the WT group data. The immunoblotting analyses were done for five randomly selected animals from each group.

### Multiplex measurement of IGF-1, p70S6K, and p-p70S6K levels

Bio-Rad Bio-Plex Luminex 200 multiplex assay system (Bio-Rad) was used to determine the levels of specific proteins of the insulin-like growth factor 1 (IGF-1)/Akt pathway (IGF-1, p70S6K, and p-p70S6K), with actin as a reference. For the analysis, 10 µg of skeletal muscle lysate protein was used. The analysis procedure was performed according to 'manufacturer's recommendations. Data were collected and processed using the Bio-Plex Manager software (Bio-Rad). The results were normalized to the amount of actin in the samples.

### Proteomic analysis

In some experiments, protein levels were determined using liquid chromatography-MS^3^ spectrometry (LC–MS/MS) at the Thermo Fisher Center for Multiplexed Proteomics (Department of Cell Biology, Harvard Medical School, Cambridge, MA, USA). The samples were prepared as previously described^39^. Peptide fractions were analyzed using an LC-MS^3^ data collection strategy and an Orbitrap Fusion mass spectrometer (Thermo Fisher Scientific Inc., Waltham, MA, USA). Principal component analysis (PCA) was performed using the R Statistical Software (Foundation for Statistical Computing, Vienna, Austria), a free software environment for statistical computing and graphics to visualize the similarities and differences between the studied groups.

### Blood sampling and creatine kinase (CK) measurements

After euthanasia, the blood was taken directly from the heart, collected into EDTA-containing tubes and centrifuged at 2000×*g* for 10 min at 4 °C. CK activity was measured in plasma samples at 37 °C using a RANDOX CK-NAC assay (cat. no. CK522, Randox Laboratories Ltd., Crumlin, UK) according to the 'manufacturer's instructions. The CK activity is expressed as U/L.

### Data analysis

Statistical analyses were performed using the software package Statistica v. 13.0 (StatSoft Inc., Tulsa, OK, USA). The results are expressed as the mean ± standard error of the mean (SEM). The normality of distribution and similarity of variances were tested to determine which statistical test should be used. The differences associated with disease progression and between ALS and WT groups were analyzed using one-way analysis of variance (ANOVA) or Kruskal–Wallis test. If a difference was detected in these test models, the significance level was then determined using 'Tukey's post-hoc test. To verify the significance of small, swim training-associated changes (ONSET untrained vs. trained and TERMINAL untrained vs. trained), 'Student's *t*-test or Mann–Whitney U test was used. The results were considered statistically significant at *p* < 0.05.

## Supplementary Information

Supplementary Figures.

## Data Availability

The data that support the findings of this study are available from the corresponding authors.
