# Supplementary material for: Swim training affects Akt signaling and ameliorates loss of skeletal muscle mass in a mouse model of amyotrophic lateral sclerosis
Source: Sci Rep. 2021 Oct 22;11:20899. doi: 10.1038/s41598-021-00319-1 (PMC8536703; doi:10.1038/s41598-021-00319-1)
Supplement: Supplementary file 1 — Supplementary Figures. [file 41598_2021_319_MOESM1_ESM.pdf]

Fig. S1 Sample picture with the total amount of protein on the membrane.

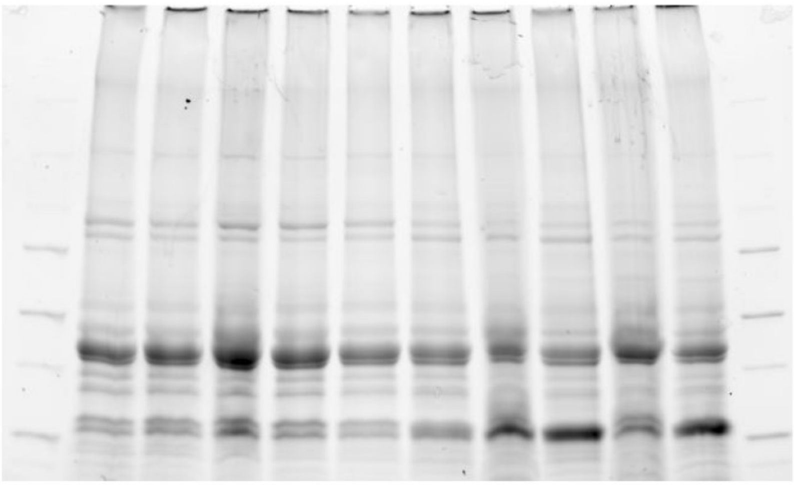

Changes in protein levels were assessed by densitometry of the immunoreactive bands and normalized to the total amount of protein in the samples transferred onto the membrane.

Fig. S2 Proteome analysis of IGF-1 and Akt levels in ALS BEFORE and TERMINAL trained and untrained groups.

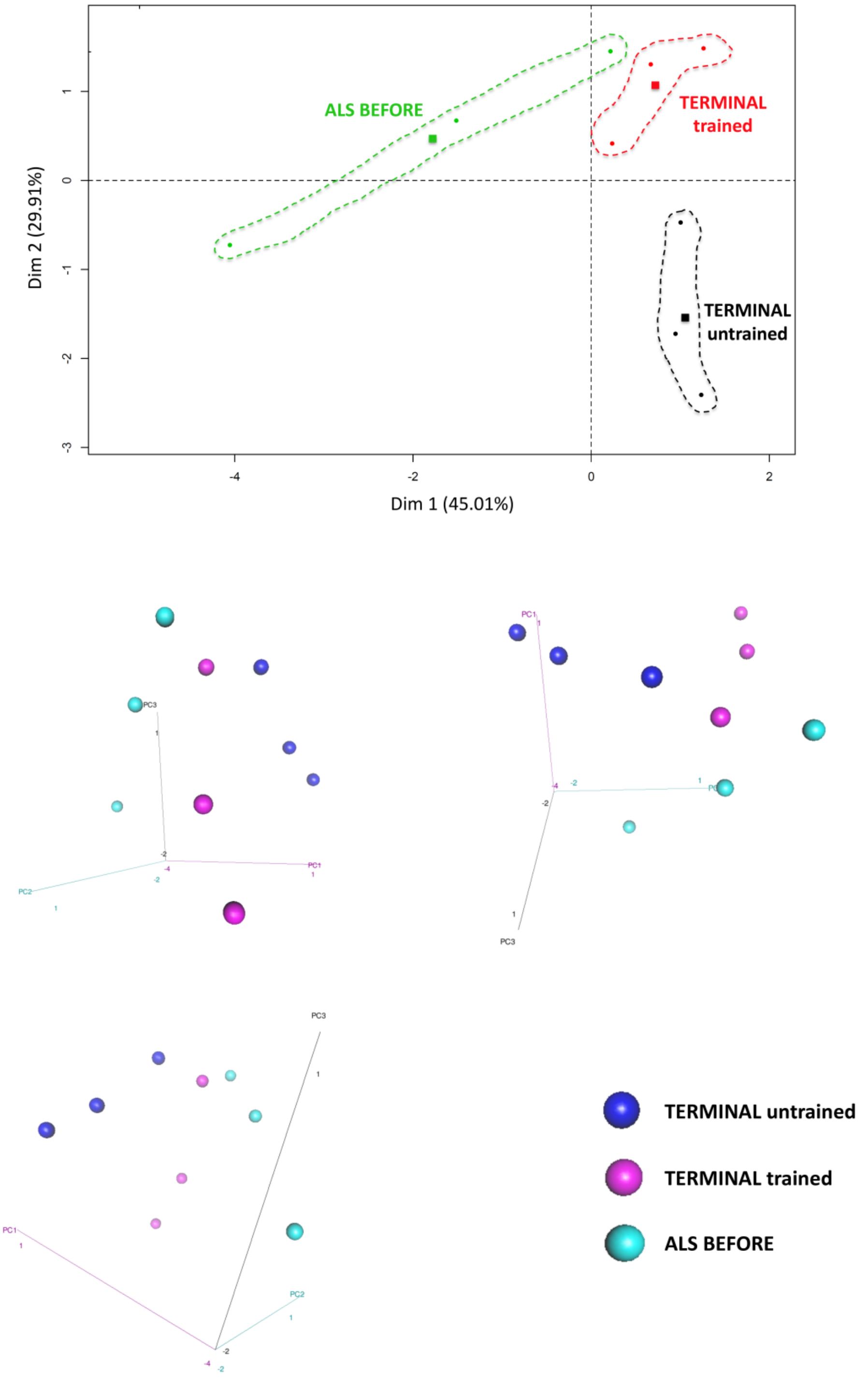

Fig. S3 Effects of ALS disease progression and swim training on the Atrogin-1 protein level (a) and original blot of the Atrogin-1 protein level (b).

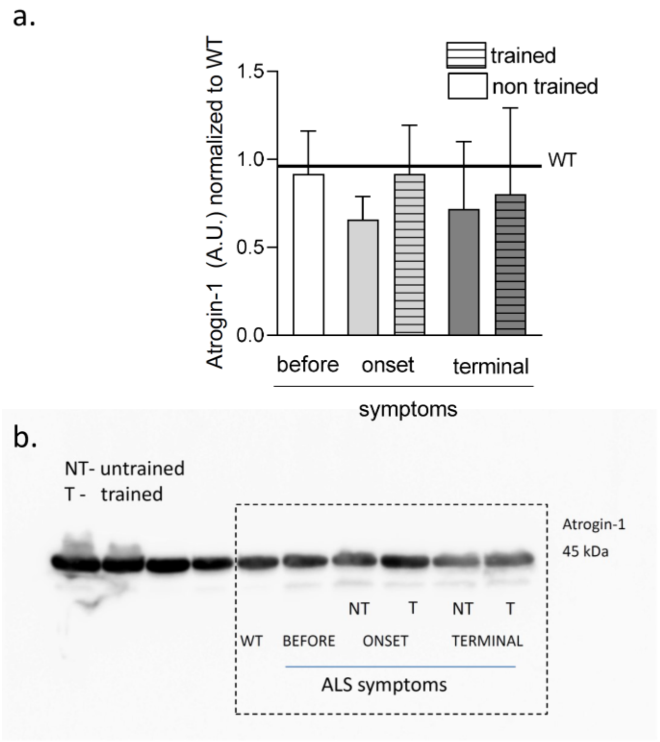

The data are presented as the means ± SEM (n = 5 in each group).

Fig. S4 Proteome analysis of FOXO3a, Atrogin-1, and MuRF1 levels in ALS BEFORE and TERMINAL trained and untrained groups.

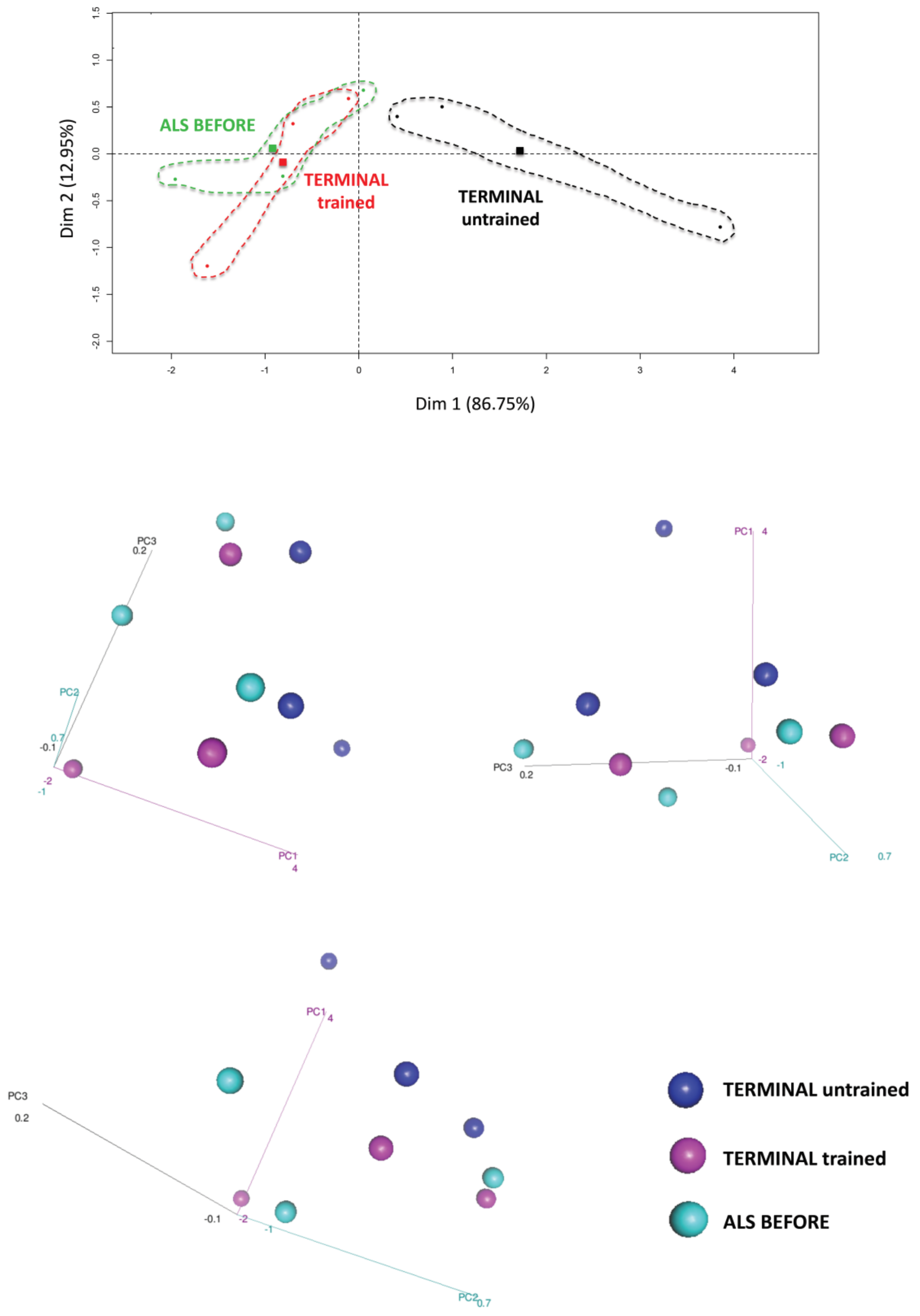

Fig. S5 Proteome analysis of mTOR and p70S6K levels in ALS BEFORE and TERMINAL trained and untrained groups.

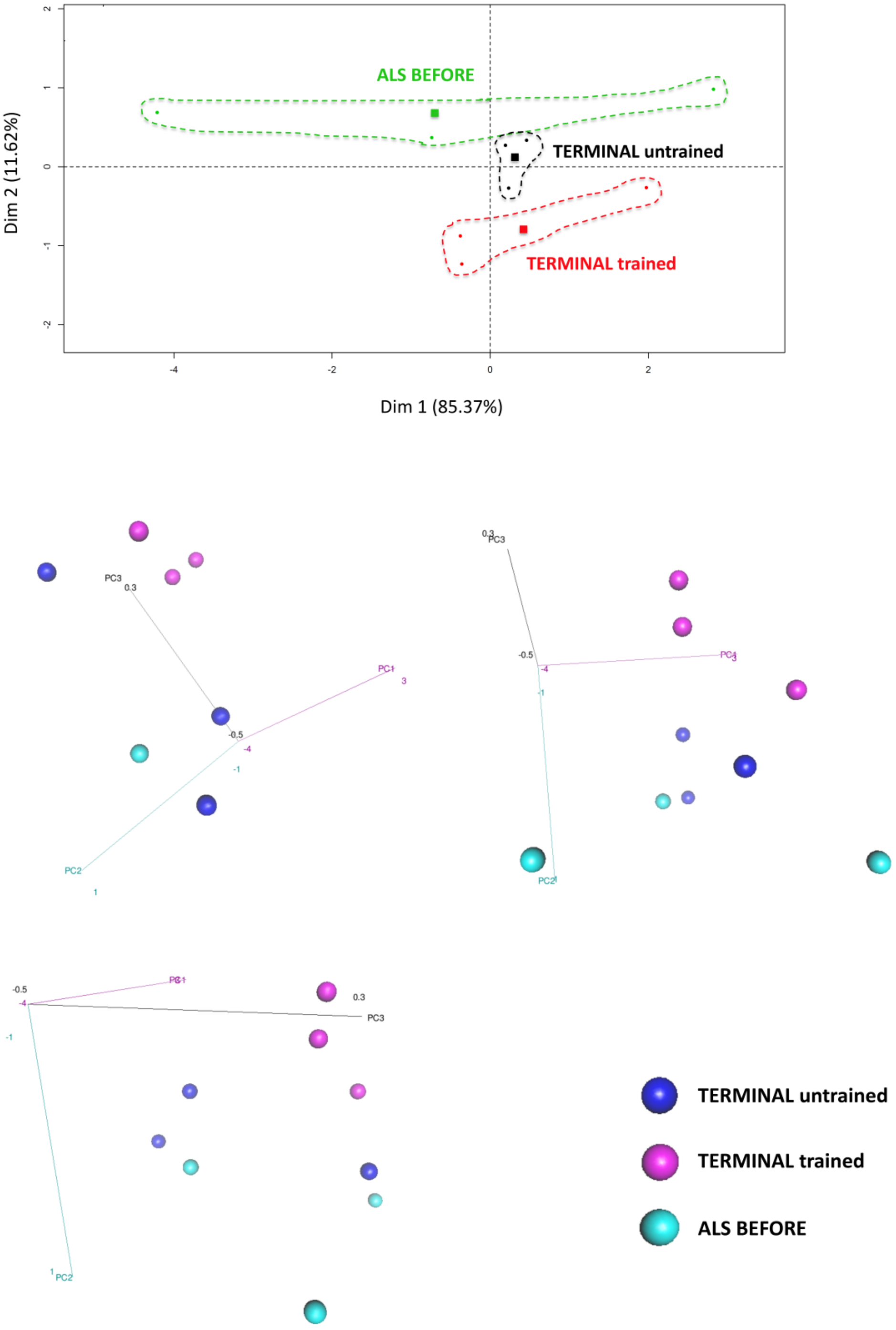

Fig. S6 Original blot of the Akt protein level.

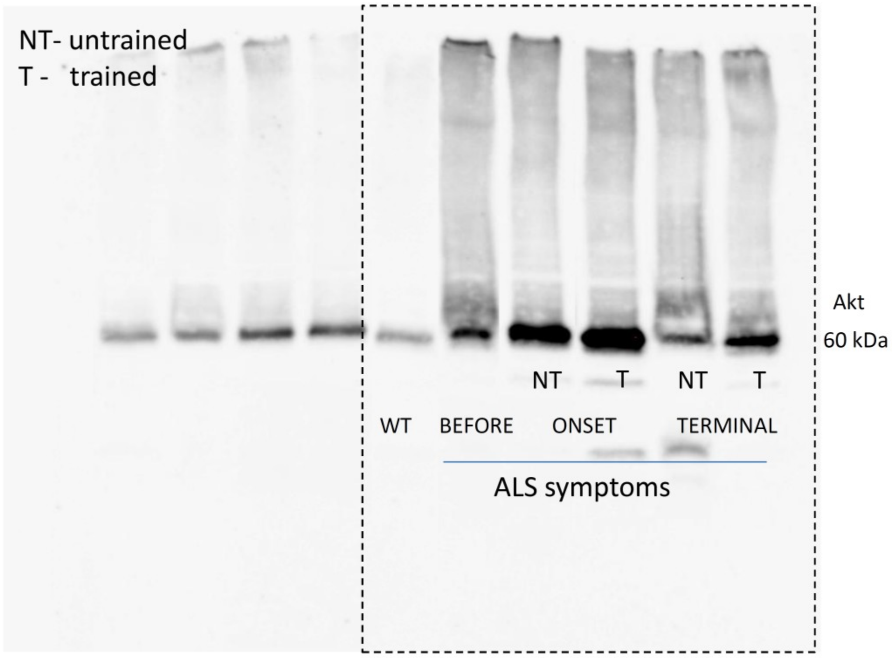

Marked square is the area included in the analysis.

Fig. S7 Original blot of the p-Akt protein level.

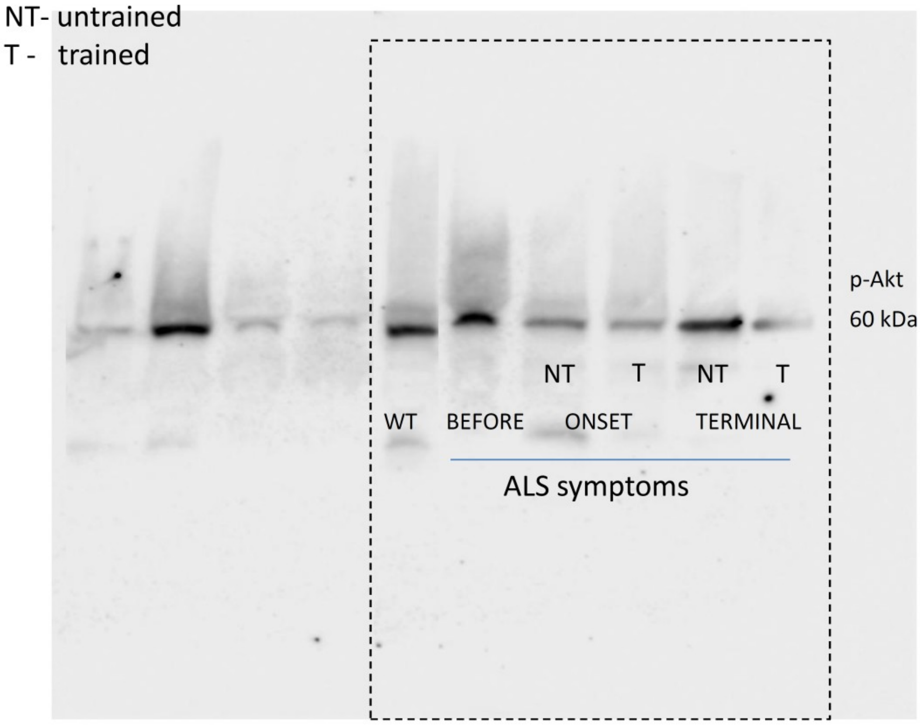

Marked square is the area included in the analysis.

Fig. S8 Original blot of the FOXO3a protein level.

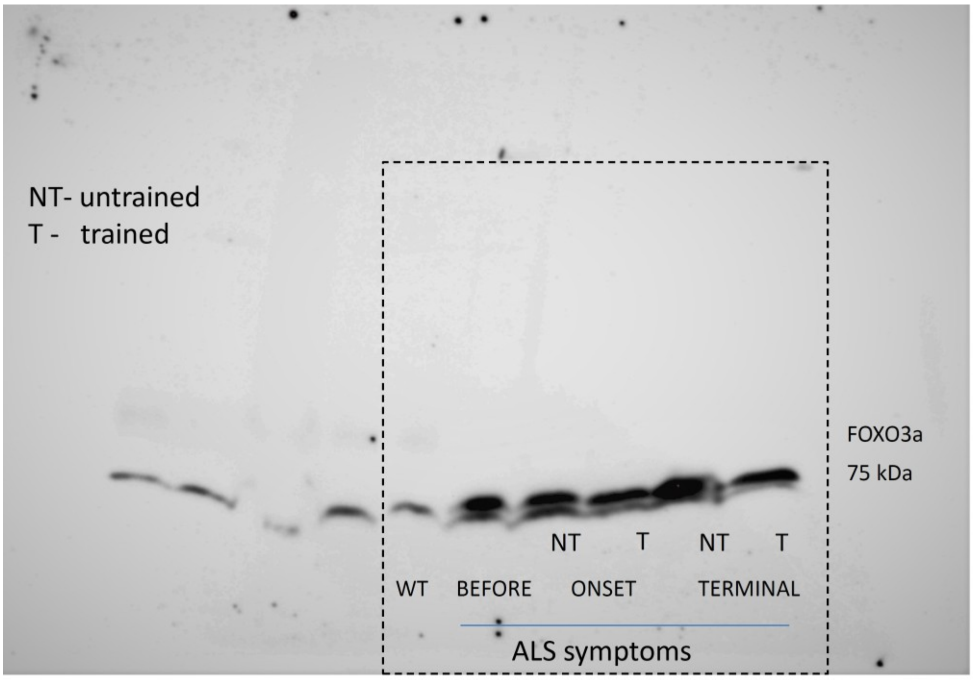

Marked square is the area included in the analysis.

Fig. S9 Original blot of the p-FOXO3a protein level.

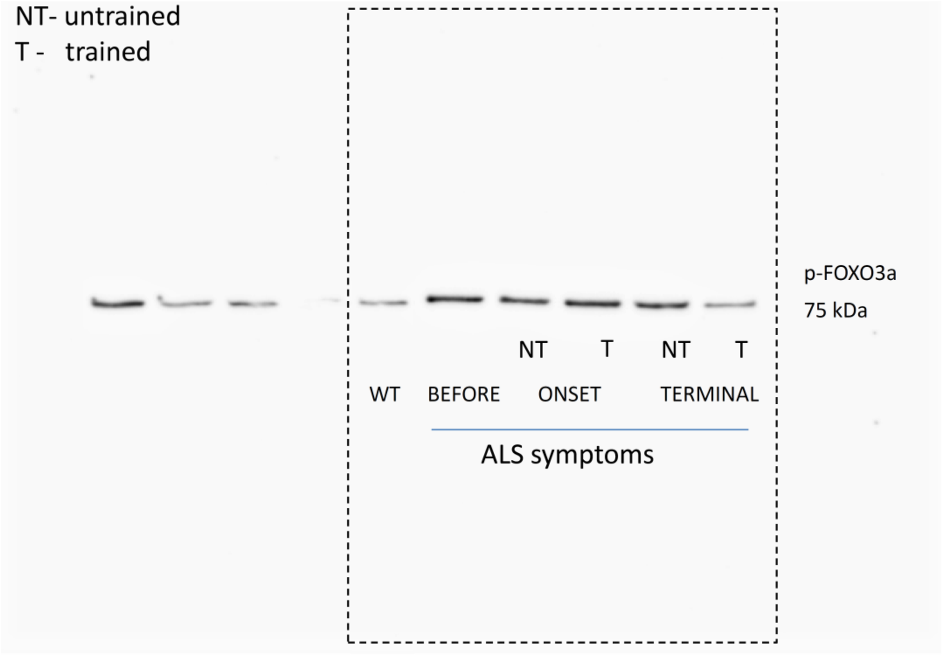

Marked square is the area included in the analysis.

Fig. S10 Original blot of the MuRF-1 protein level.

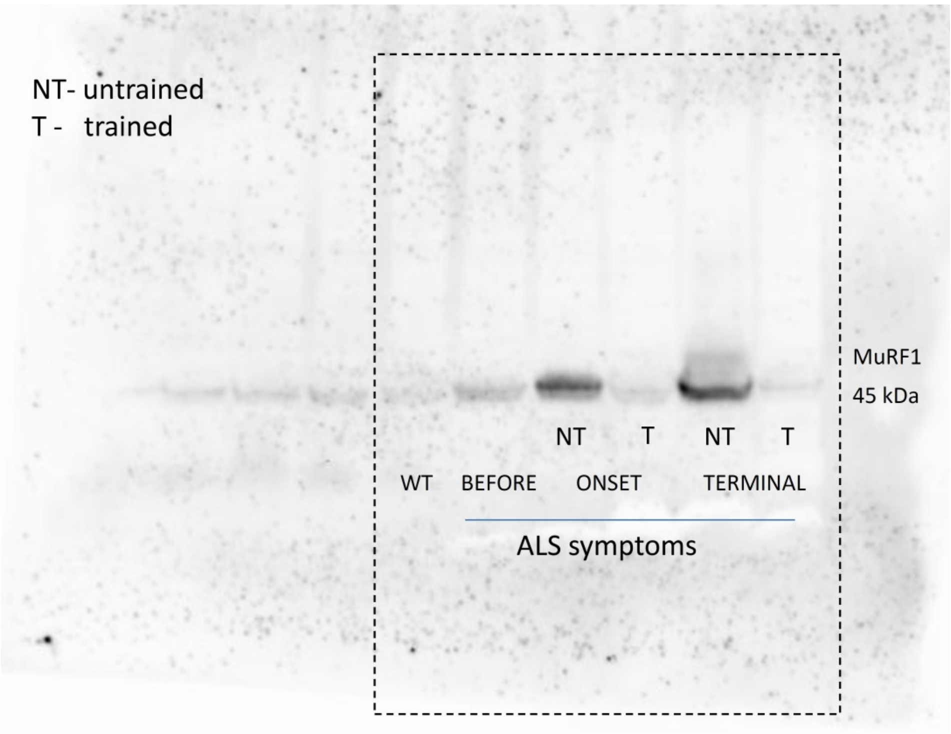

Marked square is the area included in the analysis.

Fig. S11 Original blot of the mTOR protein level.

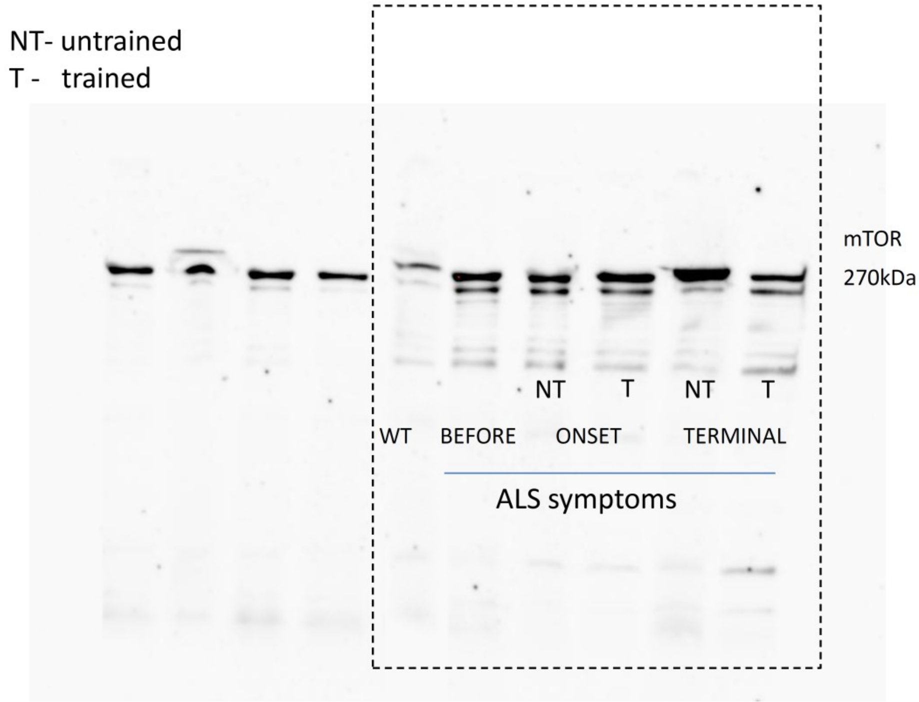

Marked square is the area included in the analysis.
